# Contralateral Pneumothorax after the Implantation of a Dual Chamber Pacemaker

**Published:** 2017-08-30

**Authors:** Cyrus M. Munguti, John M. Eliveha, Freidy A. Eid

**Affiliations:** 1 University of Kansas School of Medicine-Wichita, Department of Internal Medicine; 2 Cardiovascular Care, PA, Wichita, KS

**Keywords:** right atrium, contralateral pneumothorax, artificial cardiac pacemaker

An 85-year-old male presented to the primary care clinic with a one-month history of excessive fatigue and one episode of almost fainting. An electrocardiogram showed Mobitz II second degree heart block with a pulse rate of 36 beats per minute. He was admitted and cardiology was consulted for further work-up and management. His past medical history was significant for diabetes mellitus, hypertension, hyperlipidemia, hypothyroidism, and peripheral vascular disease. Initial laboratory tests showed a normal thyroid profile, complete blood count, liver panel, and renal function panels. His troponin was <0.04 u/dl. With no reversible causes of heart block identifiable, he was scheduled for a permanent pacemaker insertion.

He had a dual chamber Medtronic permanent pacemaker placed and remained admitted for overnight observation. The patient complained of substernal chest pain the next day but no shortness of breath. A CT scan showed a pacemaker lead perforating the right atrial myocardium causing right pneumothorax and slight mediastinal shift to the left (Figures 1 and 2). Interrogation of the pacemaker revealed the atrial lead was not capturing and had high impedance. The pacemaker lead was pulled and repositioned in a different location in the right atrium. The patient had a chest tube with underwater seal drainage with resolution of pneumothorax.

## Discussion

Acute complications of transvenous insertion of pacemakers and dual chamber implantable cardioverter defibrillators (ICD) are rare but often serious when they occur. The reported incidence of right ventricular perforation is 0.6 – 6%.0,0 Contralateral pneumothorax is one such rare complication with a reported incidence of 1%.^3^,^4^ The reported risk factors for perforation included steroid use, use of a helical screw-in lead, and use of transvenous temporary pacing in one series.^5^ The diagnosis is made when, at a minimum, the tip of a passive fixation lead or the screw of an active fixation lead passes through the myocardium and extends into the pericardial cavity.^6^ The presentation of myocardial perforation is variable, large pericardial effusions and tamponade is observed less than anticipated, perhaps due to a combination of slowed leakage from the low pressure chamber (right atria and ventricle), self-sealing properties of the ventricle wall by muscle contraction, fibrosis, or by the lead itself.^7^,^8^ The management of lead perforation is not standardized and includes lead repositioning or lead extraction for patients with severe symptoms.^9^ Our patient had clinically significant pneumothorax and had to have a chest tube placement. Repositioning of the lead was sufficient in this case and no recurrence of pneumothorax was noted upon follow-up.

## Figures and Tables

**Figure 1:**
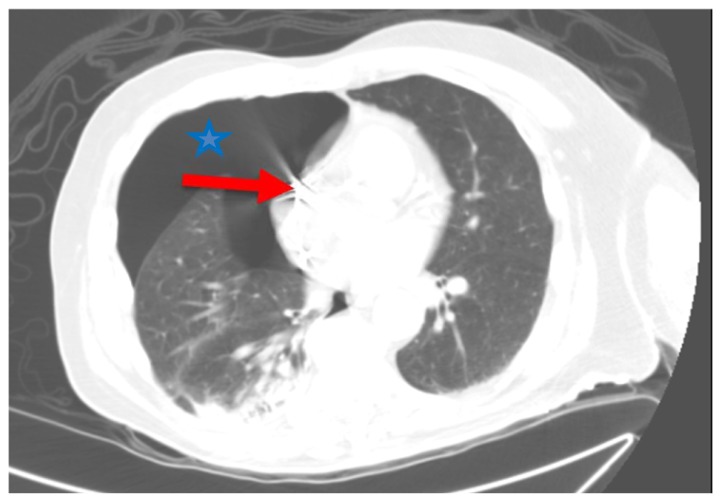
CT scan of the chest showing right-sided pneumothorax (blue star) and the tip of pacemaker lead perforating the myocardium (red arrow).

**Figure 2:**
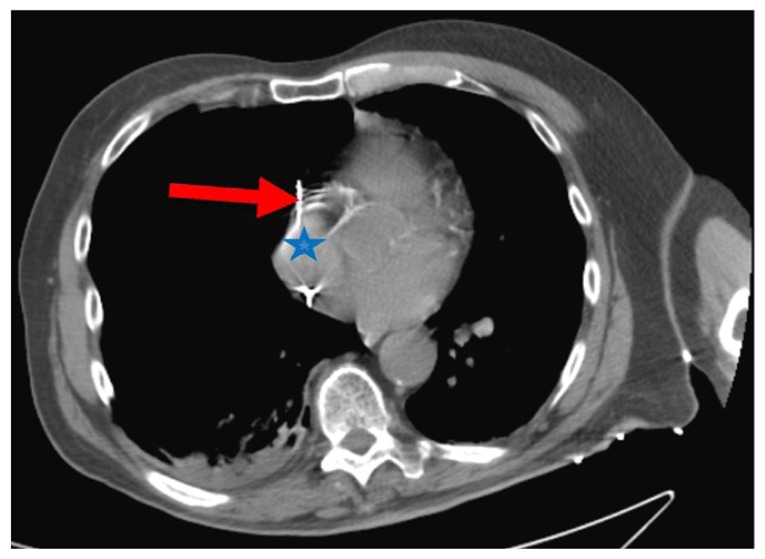
CT scan of the chest showing the tip of the pacemaker lead (red arrow) perforating through the right atrium (blue star).
